# A Narrative Review of Psychobiotics: Probiotics That Influence the Gut–Brain Axis

**DOI:** 10.3390/medicina60040601

**Published:** 2024-04-05

**Authors:** Laima Ķimse, Aigars Reinis, Lāsma Miķelsone-Jansone, Sandra Gintere, Angelika Krūmiņa

**Affiliations:** 1 Institute of Food Safety, Animal Health and Environment “BIOR”, 1076 Riga, Latvia; 2 Department of Biology and Microbiology, Riga Stradins University, 1007 Riga, Latvia; 3 Faculty of Medicine, Riga Stradins University, 1007 Riga, Latvia; 4 Department of Family Medicine, Riga Stradins University, 1007 Riga, Latvia; 5 Department of Infectology, Riga Stradins University, 1007 Riga, Latvia

**Keywords:** psychobiotics, probiotics, nervous system, gut microbiome, mental health, neurological disorders

## Abstract

Mental health disorders and dementia have become a serious public health concern, with a heightened frequency of diagnoses observed in the wake of the global COVID-19 pandemic. Psychobiotics, a novel area of research at the intersection of microbiology and neuroscience, explore the potential of probiotics to influence the nervous system and mental health outcomes. This review explores the intricate mechanisms by which psychobiotics interact with the gut–brain axis, shedding light on their effects on mood, cognition, and the stress response. Through a comprehensive analysis of the current literature and recent advancements, we discuss the therapeutic potential of psychobiotics in various mental health disorders, including depression, anxiety, and neurodegenerative diseases like dementia. The findings from this research highlight the promising potential of psychobiotics as innovative interventions in mental health treatment. Further investigation into their mechanisms of action and clinical applications is warranted to fully realize their therapeutic benefits.

## 1. Introduction

According to the World Health Organization (WHO), prior to the pandemic, approximately 970 million people worldwide were living with mental health disorders (31% with anxiety disorders, 28.9% with depressive disorders). The prevalence of mental disorders has remained stable, approximately around 13% [1]. More than 55 million people worldwide are affected by dementia. Dementia ranks as the seventh leading cause of death and is one of the primary causes of disability and dependency among older individuals worldwide [2]. New therapeutic approaches are necessary to alleviate the burden of these disorders.

The microbiota of the human gastrointestinal tract contains trillions of microorganisms, and it takes a part in maintaining human health by being involved in different physiological processes such as immunomodulation, metabolic processes, and activation of the enteric nervous system. There is also a correlation between gastrointestinal dysbiosis and disorders of the central nervous system [3].

The gut microbiota serves essential functions, particularly in a balanced state (eubiosis). These roles include the synthesis of neurotransmitters such as gamma-aminobutyric acid (GABA), serotonin, or dopamine, the regulation of cortisol levels, and the production of short-chain fatty acids critical for intestinal epithelial integrity. However, during dysbiosis, the microbiome shifts its function, leading to an increased production of pro-inflammatory cytokines, the release of bacterial toxins, and the elimination of harmful metabolites from the diet [4].

The term “probiotic”, originating from Latin (“pro” meaning for) and Greek (“bios” meaning life), translates to “for life”. Fermented items like bread, wine, beer, kefir, and cheese have historically been commonly consumed for both nutritional and medicinal reasons [5]. Probiotics refer to foods or supplements containing non-pathogenic microbes, such as bacteria and yeast, which inhabit the gut and may offer numerous health advantages. The most used genera are *Lactobacillus* and *Bifidobacterium.* Probiotics offer potential benefits in preventing and treating a range of conditions including acute diarrhea, Crohn’s disease, cardiovascular and urogenital infections, cancer, lactose intolerance, cystic fibrosis, dental caries, and oral diseases. Moreover, the bacteria found in probiotics could play a role in preventing tooth decay and treating periodontal disease. [6]. However, if probiotics are not used over an extended period, the change in gut microbiota may be temporary and short-lived [7].

Psychobiotics are defined as live bacteria (probiotics) that, when ingested in adequate amounts, have positive effects on the nervous system through an interaction with gut bacteria [8].

Psychobiotics include both beneficial bacteria and the source of food for such bacteria (prebiotics) which in human gastrointestinal tract express their beneficial effects on emotional and cognitive health. It is known that psychobiotics possess anxiolytic and antidepressant effects, and these effects are mediated by the gut–brain axis. The consumption of psychobiotics has been shown to have a positive effect on correcting microbiota dysbiosis and alleviating specific symptoms associated with central nervous system disorders, such as insomnia, depression, diabetic neuropathy, anorexia nervosa, autism spectrum disorders, Parkinson’s disease, and multiple sclerosis [9]. The objective of this review was to summarize information on the impact of psychobiotics on symptoms associated with CNS disorders such as depression, anxiety, and dementia, along with exploring the potential mechanisms underlying these effects.

## 2. Gut–Brain Axis

A large amount of psychobiotic research is conducted using animal (rodent) studies. For example, a study where mice were raised in a sterile environment (germ-free mice) and compared to normal controls (gnotobiotic mice) in stress situations showed that the germ-free mice exhibited heightened physiological reactions to stress compared to normal controls. This study revealed the role of microbiota and the hypothalamic–pituitary–adrenal (HPA) axis, with physiological reactions manifesting as an increased release of corticosterone and adrenocorticotropic hormone in germ-free mice. Furthermore, these heightened reactions were reduced by bacterial recolonization [10]. Additionally, an increase in proinflammatory cytokines activates the HPA axis, increases permeability of the blood–brain barrier, and leads to decreased level of serotonin, which can cause psychiatric disorders such as depression [11].

The gut–brain axis can be described as a bidirectional communication network that connects the enteric and central nervous systems. It includes anatomical, endocrine, humoral, metabolic, and immune routes of communication. These routes allow the brain to impact intestinal activities, and, vice versa, allow the gut to impact mental health, mood, and cognition [12].

The psychobiotic effect of bacteria primarily occurs through three distinct mechanisms: by affecting the hypothalamic–pituitary–adrenal axis stress response and reducing systemic inflammation; by an effect on the immune system; and by producing neuroactive compounds such as neurotransmitters, proteins, and short fatty acid chains [13].

### 2.1. HPA Axis

The HPA axis is a neuroendocrine system that coordinates the response of the human body to any type of stressor. The stressor triggers the hypothalamus to release corticotropin-releasing hormone, which signals the pituitary gland to release adrenocorticotropic hormone. This hormone then stimulates the adrenal glands to produce cortisol. As a result, under chronic stress, there is cortisol overproduction, which in turn increases threat sensitivity and negative mood, and impairs memory and other cognitive functions.

There is a bidirectional connection between the gut microbiota and neuroendocrine system. The establishment of gut microbiota in early life has an impact on the brain and behavior, including the stress reaction. Vice versa, the functions of the HPA axis can impact the gut microbiota composition and enlarge the permeability of the gastrointestinal tract [13,14].

### 2.2. Immune Response and Inflammation

Commensal microbes have an impact on innate and adaptive immune responses, mainly by producing molecules that mediate host–microbial interactions. A vivid example of molecules like these is short-chain fatty acids, which are a source of energy for gut epithelial cells, induce the expansion of regulatory T cells, and modulate cytokine production. Furthermore, short-chain fatty acids regulate the maturation and functions of microglia, which are primary innate immune effector cells of the central nervous system [13,15,16]. Thus, it is no coincidence that there is relevance between gastrointestinal tract dysbiosis and changes in immune responses that include the excess production of inflammatory cytokines.

### 2.3. Neuroactive Compounds

Serotonin, a neurotransmitter, is well known for its effects in regulating behavioral and biological functions by taking part in psychological processes in the central nervous system and expressing its effects in peripheral tissues (bones and gut). Most of it is produced in the human gastrointestinal tract, and microbiota contributes to its biosynthesis from enterochromaffin cells [17].

Catecholamines, such as dopamine, norepinephrine, and epinephrine, are biogenic amines. Besides their cardiovascular effects, they play a role in cognitive processes such as learning and memory formation. The microbiota of the gastrointestinal tract takes part in generating free catecholamines in the gut lumen. It has also been reported that gut microbiota maintains uptake systems for several biogenic amines, thus being a source of luminal norepinephrine [18,19].

There have been studies that reported GABA-producing microorganisms, mostly lactic acid bacteria. For example, a study by Valenzuela et al. (2019) reported GABA production by strains of *Lactococcus lactis* and *Streptococcus thermophilus* [20]. A more recent study isolated GABA-producing bacteria (*L. brevis* strains MG5552, MG5405, MG5261, MG5522) from fermented foods, and they also exhibited anti-inflammatory activity [21]. GABA and glutamate are neurotransmitters that play important roles in the normal functioning of the brain, enhancing synaptic plasticity and neuronal excitability, thus improving cognitive functions such as memory and learning.

Short-chain fatty acids are produced by gastrointestinal tract microorganisms. This happens through the saccharolytic fermentation of carbohydrates that are not digested and absorbed in the small intestine. In addition to SCFAs’ previously mentioned effects in immunomodulation and inflammation regulation, it has also been found out that SCFAs are involved in serotonin synthesis. SCFAs induce the expression of Tph1 (tryptophan hydroxylase1) that is involved in serotonin biosynthesis from enterochromaffin cells. Thus, it validates the correlation between higher levels of SCFAs and decreased depression-like behavior and anxiety [22].

Brain-Derived Neurotrophic Factor (BDNF) is a neurotrophin that affects specific neuronal populations by improving their differentiation, viability, and proper functioning. A decrease in the levels of BDNF correlates with increased manifestations of depression and anxiety [23]. Studies show that the gut microbiota affects the expression of BDNF in the central nervous system, and this expression is observed in regions of the brain that are directly involved in the development of adequate behavioral models [24].

**Figure 1:**
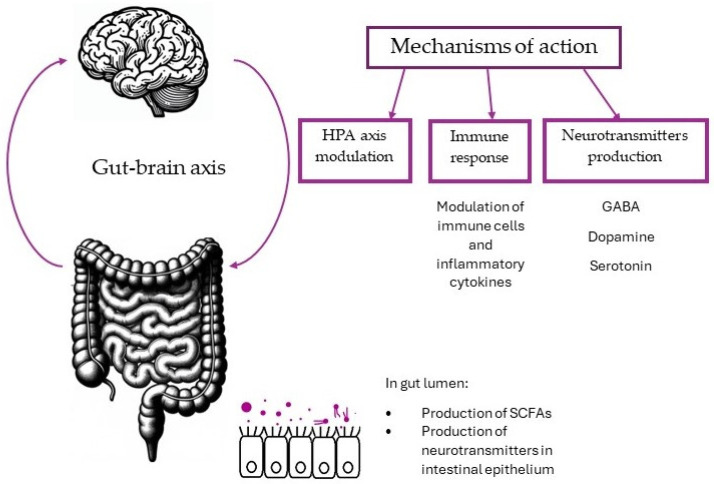
Possible mechanisms of psychobiotics.

The possible mechanisms of psychobiotics are summarized in Figure 1.

## 3. Psychobiotics in Mental Health

Depression and anxiety are highly common psychological disorders among the adult population. The mechanisms of these disorders are not yet fully interpreted, but it is known that imbalances of serotoninergic, noradrenergic, and dopaminergic processes are involved, as well as imbalances of other neuroactive compounds such as GABA and acetylcholine [25]. The cause often is related to a deficiency in the functioning of serotonin and norepinephrine; thus, first-line antidepressants are agents that prevent the reuptake of these biological amines into nerve terminals. Considering the fact that many patients develop resistance to antidepressants, and knowing that gut microbiota has an important role in regulation of these compounds, this has led to increased investigations about microbiota’s role in the treatment of these mental disorders.

Investigations have shown that there is a difference in the structure of gut microbiota in patients with depression and anxiety compared to the healthy population [26]. These findings have led to clinical trials where several specific bacterial strains have been used to evaluate the potential psychobiotic effects on individuals with mental disorders. For example, a randomized, double-blind, placebo-controlled clinical trial was conducted where 40 patients diagnosed for major depressive disorder (MDD) with irritable bowel syndrome (IBS) were given *Bacillus coagulans* MTCC 5856 for 90 days. There was statistically significant and clinically meaningful improvement in depression and IBS symptoms. The mechanism of positive effect was explained by the production of SCFAs and anti-inflammatory substances [27]. Another randomized, double-blinded, placebo-controlled clinical trial, where *Lactobacillus casei* Shirota was given to 20 male football players for 8 weeks, showed a decrease in cognitive state anxiety scores, somatic state, and perceived stress scale [28]. A randomized, placebo-controlled, double-blind clinical trial where *Lactobacillus helveticus* R0052 and *Bifidobacterium longum* R0175 were given to 110 depressed patients (81 completed the trial) showed a significant decrease in Beck Depression Inventory (BDI) score. The kynurenine/tryptophan ratio decreased significantly in the probiotic group, while the tryptophan/isoleucine ratio increased. The possible mechanism was explained by the modulation of neurotransmitter synthesis and metabolism [29]. Additional psychobiotic strains capable of alleviating symptoms of depression and anxiety are examined below (Table 1 and Table 2).

It is crucial to highlight that meta-analyses have uncovered conflicting results regarding the efficacy of psychobiotics in improving depressive symptoms. For instance, one meta-analysis examined 10 clinical trials involving a total of 1349 patients, comparing probiotic supplementation to placebo controls. While it determined that probiotics had an overall insignificant impact on mood, it also underscored several limitations in psychobiotic trials. Discrepancies in probiotic dosing, bacterial strains, and strain combinations hinder the comparability of current clinical studies. Moreover, the majority of randomized controlled trials have focused on healthy individuals, posing challenges in generalizing the findings to those suffering from depression [38].

Contrary to previous conclusions, a recent systematic review and meta-analysis, analyzing 42 trials to compare the effectiveness of antidepressants and probiotics in treating major depressive disorder, suggests that probiotics could be effective as either an adjunct or standalone therapy for managing major depressive disorder. [39].

## 4. Psychobiotics in Neurological Disorders

Parkinson’s disease is a chronic, progressive, idiopathic neurodegenerative disease characterized by both motor and non-motor system manifestations. It is the second most common neurodegenerative disease, and it is a disabling disorder that affects the middle-aged and elderly population [40,41]. Motor system manifestations appear in a fairly advanced stage of the disease, while non-motor system manifestations (including intestinal symptoms) appear years before the onset of motor system symptoms [42]. The pathogenesis of PD is based on the progressive loss of dopaminergic neurons in the substantia nigra and intracellular accumulation of α-synuclein, forming Lewy bodies and Lewy neurites [43].

There is a hypothesis that PD pathology originates from the gut and gradually spreads to the brain (the disease starts in the enteric nervous system and spreads to the central nervous system through the vagus nerve). This is supported by the evidence that depositions of α-synuclein are not only found in the gastrointestinal tract of PD patients with gastrointestinal symptoms, but also in the gastrointestinal tract of patients before they present motor system symptoms [44].

Different studies have shown dysbiosis in PD patients compared to control groups [45,46], but the results are inconsistent and difficult to compare due to varied methodology. A recent study found that people with PD might have an overabundance of a polymicrobial cluster of opportunistic pathogens, reduced levels of SCFA-producing bacteria and/or elevated levels of carbohydrate metabolizers commonly known as probiotics [47].

Constipation is one of the most common non-motor symptoms of PD. To evaluate the potential psychobiotic effects of fermented milk, in a pilot study, 40 PD patients suffering from constipation were given fermented milk containing *Lactobacillus casei* Shirota daily for 5 weeks. The results showed statistically significant improvements in stool consistency, reduced bloating, and abdominal pain [48].

In double-blind, randomized, placebo-controlled study, 72 PD patients were given multistrain probiotic capsules (*E. faecium, L. acidophilus, L. paracasei, L. rhamnosus, B. longum, B. bifidum, L. reuteri*) for 4 weeks. Spontaneous bowel movements (per week) increased after treatment with probiotics but decreased in the placebo group. There were also improvements in stool consistency and in quality of life [49].

In a randomized, double-blind, placebo-controlled clinical trial, probiotics (*Lactobacillus acidophilus, Bifidobacterium bifidum, Lactobacillus reuteri, and Lactobacillus fermentum)* were given to 60 patients with PD, daily for 12 weeks, to assess the impact of probiotics on movement and metabolic parameters in PD patients. The consumption of probiotics decreased the score on The Movement Disorders Society-Unified Parkinson’s Disease Rating Scale (a scale that measures the severity and progression of PD). There were also changes in the metabolic parameters: reduction in high-sensitivity C-reactive protein and malondialdehyde levels, increase in glutathione levels, and improvements in insulin function [50].

In one experimental study, 16 male mice were given daily probiotics (consisting of *Bifidobacterium bifidum*, *Bifidobacterium longum*, *Lactobacillus rhamnosis*, *Lactobacillus rhamnosus* GG, *Lactobacillus plantarum* LP28, and *Lactococcus lactis* subsp. Lactis) for 16 weeks. The study found that the daily administration of probiotics significantly improved gait, balance, and coordination [51].

Alzheimer’s disease is a chronic neurodegenerative disease which progresses to memory and cognitive decline. It is the most common form of dementia. The most recognized features of AD pathology are amyloid plaques (extracellular accumulations of abnormally folded amyloid beta proteins) and neurofibrillary tangles (paired helical filaments consisting of hyperphosphorylated tau). Their deposition leads to the loss of synapses and nerve cells, leading to atrophy in the brain [52]. While many studies have been performed looking into the causes of Alzheimer’s disease, the underlying mechanisms remain unclear. Investigations suggest that changes in the gut and blood–brain barrier permeability due to microbiota dysbiosis might influence the onset of AD. Also, the gut microbiome can produce substances (amyloids and lipopolysaccharides) that affect signaling pathways and lead to the production of pro-inflammatory cytokines linked to AD. Additionally, microbiota dysbiosis might trigger inflammation associated with AD [53].

Several studies on mice have been performed to understand how probiotics impact AD. One experimental study found improved cognitive dysfunction and a suppressed expression of inflammation in AD male mice given *Bifidobacterium breve* strain A1 [54]. In another experimental study, 48 D-Galactose-induced AD rats were given *Lactobacillus plantarum* for 60 days; results showed the amelioration of cognition deficits and restoration of acetylcholine levels in the cortex and hippocampus [55].

In another experimental study, a mixture of probiotics (*Lactobacillus acidophilus*, *L. fermentum*, *Bifidobacterium lactis*, and *B. longum*) was given to 60 male rats (injected with β-amyloid) for 8 weeks. Results showed improved spatial memory and improvements in oxidative stress biomarkers [56]. In a recent experimental study, *Lactobacillus mucosae* and *Bifidobacterium longum* were given to 30 male mice; this combination showed alleviated cognitive decline and neuroinflammation. They also alleviated colitis and gut dysbiosis [57].

Some clinical studies have investigated the beneficial properties of probiotics in AD patients. In randomized, double-blind, controlled clinical trial, 79 AD patients were given a combination of probiotics (*Lactobacillus acidophilus*, *Bifidobacterium bifidum*, and *Bifidobacterium longum*) and selenium for 12 weeks to determine the effects on cognitive function and metabolic status. Compared with other groups (only selenium and placebo), probiotic and selenium co-supplementation resulted in a significant increase in the mini-mental state examination score (MMSE). There were also improvements in the metabolic profiles (reduced hs-CRP, increased total antioxidant capacity, increased GSH, reduced insulin levels, and reduced triglycerides and total-/HDL-cholesterol) [58]. In another randomized, double-blind, controlled clinical trial, probiotic milk containing *Lactobacillus acidophilus*, *Lactobacillus casei*, *Bifidobacterium bifidum*, and *Lactobacillus fermentum* was given to 60 AD patients for 12 weeks to assess probiotic effects on cognitive function and metabolic status. The probiotic group showed a significant improvement in the MMSE score [59].

In uncontrolled clinical study, 13 AD patients were given probiotic-fermented milk supplementation (kefir) for 90 days. The majority of patients exhibited improvements in memory, visual/spatial abilities, and language skills [60].

While these studies show promising potential in terms of alleviating symptoms associated with stress, anxiety, depression, and dementia, it is important to acknowledge that they are accompanied by certain limitations and shortcomings. These limitations and shortcomings encompass a wide array of factors related to study design, methodology, and interpretation. It is essential to give them thoughtful consideration in order to effectively propel advancements in the field.

One significant limitation lies in the clinical trial design. Many studies suffer from methodological constraints such as small sample sizes, short follow-up periods, and inadequacies in blinding and randomization procedures. These flaws undermine the robustness of findings and hinder the extrapolation of results to broader populations.

Another challenge is the heterogeneity in study design. Variations in bacterial strains, dosages, treatment durations, and administration methods contribute to significant disparities across studies. Consequently, comparing results and drawing definitive conclusions regarding the efficacy of probiotics for specific disorders becomes challenging.

Furthermore, there is an absence of consistency in probiotic research. Discrepancies in how interventions are reported and outcomes are assessed impede replication efforts and hinder effective meta-analyses, thereby limiting the reliability of findings.

Moreover, the mechanisms through which psychobiotics impact mental health remain poorly understood. Further investigation is necessary to unravel the intricate interactions among gut microbiota, the gut–brain axis, and neural pathways, which can pave the way for more targeted interventions.

Additionally, psychobiotic effects may vary among individuals due to differences in gut microbiota composition, genetic makeup, diet, and lifestyle factors. Comprehensive studies involving diverse populations are essential to ascertain the breadth and consistency of these effects across demographics.

The majority of existing studies on psychobiotics are short-term and focus on immediate outcomes, thus warranting longitudinal studies to assess sustained effects over time and investigate potential long-term benefits or risks associated with their use.

Despite initial evidence suggesting therapeutic benefits, further robust clinical trials are necessary to confirm the effectiveness, safety, recommended dosages, and possible side effects of psychobiotics in clinical practice.

Furthermore, ensuring the quality, purity, and uniformity of psychobiotic formulations is crucial for dependable results. Establishing standardization protocols and quality control procedures is imperative to facilitate study comparisons and ensure the effectiveness and safety of psychobiotic products.

Also, extrapolating results from animal studies to human conditions presents inherent challenges due to biological disparities, complexities of human diseases, ethical restrictions, and variations in dosage and treatment regimens. These challenges include the limited generalizability of findings, difficulties in translating interventions into effective human treatments, and the potential distortion of interpretation due to publication bias. Combining evidence from both animal and human studies is essential to comprehensively understand disease mechanisms and develop effective therapies.

In conclusion, addressing these limitations and shortcomings is paramount for advancing our understanding of psychobiotics and harnessing their full potential in mental health interventions.

## 5. Side Effects of Psychobiotics

Most probiotics available today originate from either fermented foods or the naturally occurring microbes found in a healthy individual, and they have been utilized in various products for many years. Because of their prevalence in fermented foods and their natural presence in the human body, *Lactobacillus* strains are deemed to have a low risk of causing illness, as evidenced by the minimal occurrence of associated infections. Likewise, *Bifidobacterium* species exhibit a similar level of safety [61].

Probiotics, generally regarded as safe for most individuals, may occasionally lead to side effects. These include mild gastrointestinal discomfort (bloating, flatulence, and diarrhea), particularly during the initial stages of probiotic supplementation as the gut microbiota adjusts. In some cases, individuals may experience allergic reactions to specific strains of bacteria, resulting in rash, itching, and swelling. These symptoms are rare and should resolve on their own after the discontinuation of probiotics [62]. Probiotics may lead to more severe adverse effects such as bacteremia, fungemia, and endocarditis, particularly among vulnerable populations including infants, the elderly, and individuals with compromised immune systems [63].

## 6. Conclusions

Psychobiotics have the potential to positively affect mental health by modulating the gut–brain axis. These beneficial bacteria interact with the gut microbiota and generate compounds that can signal to the brain, potentially enhancing mood, cognition, and the stress response. While studies indicate that psychobiotics could offer therapeutic benefits for conditions like depression, anxiety, and neurodegenerative diseases such as dementia, the precise mechanisms of action are still under investigation. Therefore, addressing the identified limitations and shortcomings in research is crucial to fully harness the potential of psychobiotics and to advance their integration into clinical practice for the benefit of individuals struggling with mental health disorders. Further explorations of their mechanisms and long-term effects, along with standardization and quality assurance measures, will contribute to establishing psychobiotics as valuable tools in the mental health treatment toolkit.

## Figures and Tables

**Table 1:** Review of clinical trials on probiotics with psychobiotic properties that may be beneficial in patients with depression and anxiety.

Reference	Study Type, Population, Duration	Psychobiotic	Effects of Psychobiotic Therapy	Possible Mechanisms
[30]	A within-subjects design (a repeated measure, placebo-controlled design); 22 healthy male volunteers, 8 weeks	*Bifidobacterium longum* 1714 (dosage—1 × 109 CFU/day)	Cortisol levels and anxiety experienced during the cold pressor test (SECPT) were reduced. Decrease in daily reported stress. Subtle improvements in hippocampus-dependent visuospatial memory performance and enhanced frontal midline electroencephalographic mobility.	Brain-Derived Neurotrophic Factor synthesis through vagal activation
[31]	A randomized, double-blind, placebo-controlled study, 44 adults with IBS and diarrhea or a mixed-stool pattern (based on Rome III criteria) and mild to moderate anxiety and/or depression, 6 weeks	*Bifidobacterium longum* NCC3001 (dosage—1 × 1010 CFU/g)	Reduced depressions scores on Hospital Anxiety and Depression Scale. Reduced response to negative emotional stimuli in multiple brain areas. Increase in quality-of-life score.	Distribution of neuroactive compounds through vagal signaling
[32]	An open-label, prospective, randomized trial, 40 antidepressant-treated patients, 8 weeks	*Clostridium butyricum* MIYAIRI 588 (dosage—60 mg/day)	Used in combination with antidepressants shows a positive effect in the treatment of treatment-resistance depressive disorders. A 50% reduction in depression and anxiety scores (HAMD-17; BDI, BAI)	Neuroprotective—neurogenesis, antioxidation, mitigating glutamate excitotoxicity, and directly regulating proinflammatory agents.
[33]	A double-blind, placebo-controlled trial, 49 healthy medical students, 8 weeks	*Lactobacillus casei* Shirota (dosage—100 mL of a fermented beverage containing more than 1 × 109 CFU/mL/day)	Decrease in stress-associated physical symptoms (abdominal, cold symptoms). Preserves the diversity of gut microbiota—significantly higher numbers of species.	Modulation of HPA axis. Increased serotonin synthesis

**Table 2:** Review of rodent models on probiotics with psychobiotic properties that may be beneficial in patients with depression and anxiety.

Reference	Study Type, Population, Duration	Psychobiotics	Effects of Psychobiotic Therapy	Possible Mechanisms
[34]	Experimental study, 60 male rats, 8 weeks	*Faecalibacterium prausnitzii* ATCC 27766	Prevention and positive therapeutic effects on anxiety and depression-like behavior. Reverse impact of chronic unpredictable mild stress (CUMS). Higher levels of SCFAs in the cecum, and higher levels of Il 10 in plasma. Reduced the enhanced level of circulating corticosterone, CRO, and cytokine IL-6 levels.	Normalized activity of the HPA axis. Anti-inflammatory effects.
[35]	Experimental study, 40 male mice, 5 weeks	*Bifidobacterium breve* CCFM1025	Reduction in depression and anxiety behaviors	Regulation of the HPA axis and increased expression of BDNF. Increase in serotonin levels
[36]	Experimental study, 35 male rats, 8 weeks	*Lactobacillus plantarum* ATCC 8014	Serum and amygdala antioxidant markers (SOD, GPx, MDA, and TAC) were significantly increased. Symbiotic consumption with inulin led to a significant increase in the amygdala levels of BDNF and serotonin in diabetic rats. Positive effects on the elevated plus maze and forced swimming tests	Modulation of HPA axis through a reduction in oxidative stress. Increase in levels of serotonin and BDNF in the amygdala
[37]	Experimental study, 48 male mice, 2 weeks	*Lactobacillus plantarum* 90sk and *Bifidobacterium* adolescentis 150	Reduction in depressive-like behavior in the forced swimming test (effect similar to that of fluoxetine)	The bacteria’s ability to produce GABA
